# Editorial for the Special Issue “Risk Assessment of Food Contact Materials/Articles”

**DOI:** 10.3390/toxics11030254

**Published:** 2023-03-09

**Authors:** Isabelle Séverin

**Affiliations:** Derttech “Packtox”, NUTOX Team, UMR INSERM-University of Burgundy-L’Institut Agro Dijon 1231, F-21000 Dijon, France; isabelle.severin@agrosupdijon.fr

Food packaging is made of four main materials, namely plastic, cardboard, glass and metals (aluminium and steel), as well as many other materials (wood, waxes, corks, etc.). The purpose of these materials is to ensure the protection and preservation of the product, the storage, sale and transport of foodstuffs, and communication and marketing related to the brand and regulations concerning labelling and traceability. Alongside these beneficial functions, packaging can nevertheless present a hazard to the consumer due to the existence of content/container interactions that take place, regardless of the material used, and in particular due to the fact that chemical substances can be released from the packaging when in contact with food; this is the migration phenomenon [1].

More than 12,000 individual substances have been identified for use in the manufacture of Food Contact Materials (FCMs) [2]. However, FCMs must comply with Article 3 of the European framework regulation (EC) No. 1935/2004 [3], which emphasises that, among other things, “Materials and articles shall be manufactured in compliance with good manufacturing practice so that, under normal or foreseeable conditions of use, they do not transfer their constituents to food in quantities which could endanger human health, …”. For example, during the manufacture of plastic packaging, starting substances (monomers, additives, etc.) may be used if a risk assessment has been carried out prior to their authorisation; these are known as Intentionally Added Substances (IAS). These authorised substances are then listed with conditions of use, and with restrictions on use for some of them, in order to protect consumer health (Annex 1 of Regulation (EU) No 10/2011) [4].

However, during the manufacturing process of the packaging or during post-treatment (packaging, microwave...), chemical reactions or degradation can occur, leading to the appearance of new substances that are not predictable, often difficult to identify and for which no toxicological data are available. These are called Non-Intentionally Added Substances (NIAS). NIAS may represent a large proportion of all substances that migrate into food [5,6,7] and McCombie et al. (2020) [8] have identified between 30,000 and 100,000 NIAS. The contamination of food and the associated health risk may then be underestimated due to the presence of these NIAS.

Although Regulation 10/2011 on plastics is the only regulation that requires assurance of the health risk of NIAS, all packaging is affected by their presence, including bio-based, reusable and recycled materials, which will be increasingly present on the market in the context of the circular economy. Thus, the increased use of recycled materials and the reuse of packaging may lead to an increase in unintentionally present contaminants, which may migrate to the food and expose the consumer to a cocktail of various substances [9]. Circular economy regulations (European Directive (EU) 2019/904, AGEC French law n°2020-105) [10,11], which urge states to reduce the amount of packaging as well as to increase recycling and reuse, are forcing manufacturers to question the compliance of these new packaging materials, which must be of the same sanitary quality as virgin materials. The safety of food contact materials/articles (FCMs/FCAs) is an important issue, accentuated by these new regulations on the circularity of packaging and the end of single-use plastic packaging.

However, unlike IAS, and even though plastic Regulation (EU) No 10/2011 requires a risk assessment of NIAS in the same way as IAS, this remains difficult. There is no harmonised guideline for an appropriate risk assessment, and the classical approach based on the identification and quantification of substances present in a migrate and their toxicological assessment is not conceivable due to time and cost constraints. Furthermore, technically, the chemical analysis of an extract/migrate is rarely exhaustive [12,13,14].

Professional associations (Food Packaging Forum [15], ILSI Europe [16] and management agencies [17,18,19] encourage global safety assessment for chemically complex mixtures containing unknown substances such as FCA by combining physical-chemical methods with reliable bioassays as a quick and cost-effective strategy. The European Commission also supported a proactive evolution of the regulation to include bioassays along with analytical testing and migration modelling techniques. They have already demonstrated their efficiency in identifying hazards and the mode of action of pure substances. Chemical analyses cannot assess the cocktail effect of FCA, whereas testing the whole migrate with bioassays can [20,21,22]. Bioassay methodology for the risk assessment of FCM/FCA, NIAS has already been reviewed by ILSI (2016) [12].

It is therefore essential to develop protocols to ensure the safety of future packaging by identifying the unknown substances or by testing the toxicity of the complex mixture of substances migrating from FCMs/FCAs. This is the challenge taken up by the following articles published in this Special Issue.

Miralles et al. developed a fast and automated approach to tentatively identify and assess the risk of unknown substances in plastic FCMs using gas chromatography–high-resolution mass spectrometry (GC-HRMS). They applied this approach to recycled low density polyethylene (LDPE) and identified 83 substances, most of which were additives used in various plastic applications. Based on the threshold of toxicological concern approach, the authors found that the release of the identified substances did not pose a risk. Furthermore, further studies on unidentified substances and potential mixture toxicity are needed [23].

Plant fiber/plastic composites (PPCs) are considered as an economical and environmentally friendly alternative to traditional petrochemical-based plastics for food contact products. However, Zhang and Weng pointed out that PPCs may pose food safety risks due to the migration of hazardous substances during the production process. The authors recommended that systematic research on migration methods and safety assessments are needed to address the potential safety risks of PPCs [24].

To evaluate the safety of FCMs, it is important to exclude mutagenicity and genotoxicity in migrates but current genotoxicity assays were not enough sensitive in terms of the biological positive threshold. Rainer et al. compared two commonly used formats of the Ames test, the standard preincubation Ames test and the liquid-based Ames MPF^TM^, to identify DNA-reactive genotoxic substances. They found that both formats showed high concordance for mutagenic versus non-mutagenic compound classification, but the lowest effect concentrations (LEC) of the Ames MPF^TM^ format were lower for 17 of the 21 tested known substances, indicating that this format could be preferable for the detection of complex mixtures of substances from FCM/FCA [25].

Debon et al. proposed the high-performance thin-layer chromatography (HPTLC) coupled with the planar SOS umu-C (p-Umu-C) bioassay as a promising rapid test to detect low levels of mutagens/genotoxins in complex mixtures. An effective bioactivation protocol was developed, and all tested known mutagens could be detected at low concentrations. The threshold of detection was very low compared to regulatory bioassays currently performed, such the Ames test. The p-Umu-C bioassay may become instrumental in the genotoxicity testing of mixture, such as food packaging migrates [26].

Finally, Marin-Kuan et al. combined both identification and testing and present a protocol combining data from analytics and bioassays for the risk assessment of packaging materials. This protocol includes guidance on sample preparation, migrant simulation, chemical analysis using liquid chromatography (LC-MS) and validated bioassays covering endocrine activity, genotoxicity and metabolism-related targets and it was tested through an inter-laboratory study on coating metal packaging materials [27].

To conclude, these five studies illustrate the great challenges facing FCM/FCA research currently and they can be used by regulators, industry and other stakeholders to improve the safety of FCM/FCAs.

I would like to express my gratitude to all authors for submitting their original contributions to this Special Issue and to the reviewers for their essential role in assessing the suitability of the manuscripts and improving their quality. I would like also to thank the editors of *Toxics* for their kind invitation, and in particular Evelyn Ning and, previously, Doris Sun of the *Toxics* Editorial Office for their valuable and tireless support.
